# Seroprevalence of Hepatitis E Virus Infection Among People Living With HIV in the Central African Republic

**DOI:** 10.1093/ofid/ofy307

**Published:** 2018-11-19

**Authors:** Ornella Anne Demi Sibiro, Alexandre Manirakiza, Narcisse Patrice Komas

**Affiliations:** 1 Hepatitis viral Laboratory, Institut Pasteur de Bangui, Bangui, Central African Republic; 2 Epidemiological Service, Institut Pasteur de Bangui, Bangui, Central African Republic

**Keywords:** Central African Republic, hepatitis E virus, HIV-HEV coinfection, people living with HIV, seroprevalence

## Abstract

**Background:**

Hepatitis E virus (HEV) is a waterborne virus that causes acute hepatitis in immunocompromised patients and those who are immunocompetent. Few cases of chronic HEV have been described in industrialized countries. The Central African Republic is 1 of the few countries in the world that is endemic for both HIV and HEV. The prevalence of HIV infection is estimated to be 4.9% among adults aged 15–49 years, and hepatitis E is epidemo-endemic. The aim of this study was to characterize the epidemiology of HEV infection in people living with HIV (PLHIV) in Bangui.

**Methods:**

A cross-sectional study was conducted between April and September 2015 based on answers to a questionnaire, and blood samples were collected for determination of immunological markers by enzyme-linked immunosorbent assay and molecular tests.

**Results:**

Of the 200 PLHIV included, 15 (7.5%) had the IgM that characterizes acute HEV infection; 8.9% were women, and 2.2% were men. The overall seroprevalence of IgG was 68% (48% women and 70.4% men), indicating that men are statistically significantly more exposed to HEV than women. HEV infection had no effect on transaminase or T-CD4+ lymphocyte cell levels. The origin of infection could not be identified.

**Conclusions:**

The seroprevalence of HEV is very high among PLHIV and in the general population of Bangui. This must be taken into account in studies of the risk factors of PLHIV infected with HEV.

Hepatitis E virus (HEV), first identified in 1990, is 1 of the main agents of entero-transmissible viral hepatitis, which became a major global public health problem at the beginning of the 21st century [1–3]. HEV infection is most often self-limited and asymptomatic. Currently, 4 major HEV genotypes have been identified (HEV1, HEV2, HEV3, and HEV4), all belonging to a single serotype but differing in geographical distribution and infection modality. HEV1 and HEV2 genotypes have been involved in waterborne hepatitis E outbreaks and in sporadic cases in developing countries where HEV is epidemic. In developed countries, the HEV3 and HEV4 genotypes are also endemic and responsible for sporadic autochthonous hepatitis E. It has been established that the major reservoir of these 2 genotypes, zoonotic and foodborne HEV infection, constitutes the main route of HEV transmission. The HEV3 genotype is more involved in the chronic course of hepatitis E, and cases of chronic hepatitis E are being reported more and more often [4–9]. The mechanisms leading to chronic HEV infection and the rates of mortality among pregnant women infected with HEV (≤25%) are largely unknown [10]. As cases of chronic HEV infection have been reported in immunocompromised individuals, HEV may be a significant threat to the survival of people living with HIV (PLHIV) [11–14]. Immune system failure due to HIV infection exposes the body to attack by other infectious agents that are controlled by a healthy immune system. Few studies have been done on the prevalence of HEV infection among PLHIV in resource-poor countries such as the Central African Republic [5, 6], although several outbreaks have been reported in Bangui, the capital [15–18]. The aim of this study was to estimate the seroprevalence of HEV infection among PLHIV in Bangui and to identify the risk factors for HEV infection in this population.

## METHODS

This cross-sectional study was conducted at the Viral Hepatitis Laboratory of the Institut Pasteur de Bangui on blood samples collected from PLHIV referred by physicians for their biological follow-up between April 7 and September 30, 2015. These PLHIV were supported by the HIV/AIDS program. An investigator for this study collected information from each participant with a standardized questionnaire. Finally, a total number of 200 PLHIV were recruited for the study. Sociodemographic data such as sex, age, and geographical origin (Bangui district and its suburbs) were recorded. Sera were obtained from blood samples collected in dry tubes and centrifuged at 3900 rpm for 10 minutes, aliquoted, and stored at –20°C before biological and molecular testing. Blood samples were collected in EDTA-containing tubes for determination of TCD4+ lymphocyte cells and blood count.

HEV infection was diagnosed by detection of HEV immunoglobulins (IgG and IgM) with a commercial enzyme-linked immunosorbent assay (ELISA) kit (Diagnostic Bioprobes Srl DIAPRO, Milan, Italy). The results were interpreted according to the recommendations of the manufacturer. RNA extracted with a Qiagen kit (QIAamp Viral RNA Mini Kit, QIAGEN Gmbh, Hilden, Germany) was subjected to real-time reverse transcription polymerase chain reaction (RT-PCR) amplification in the presence of the sense primers F (TaqHEV-F, GCCCGGTCAGCCGCTCTGG), antisense R (TaqHEV-R, CTGAGAATCAACCCGGTCAC), and probe S (TaqHEV-S, FAM-CGGTTCCGGCGGTGGTTTCT-TAMRA) [19]. The size of amplicon need to confirm the presence of the virus in the sample was 89 bp.

Data were analyzed with Epi Info 7 software, version 7.1.5.0. Tests to determine the frequency of distribution of each variable studied were performed. First, we analyzed the percent distribution of sociodemographic (age, sex) and biological data: TCD4+ (≤350 and >350), hepatic transaminases (normal and high), and the characteristics of well water used in households (treated, untreated, and distance between well and latrines). To define the areas of infection with HEV, we analyzed the samples according to the geographical origin of the patients within the 8 districts of Bangui. In a second step, we evaluated the association between these variables and HEV infection status. The chi-square test (or Fisher exact test) and odds ratios (ORs) with 95% confidence intervals (CIs) were used to determine the association between HEV infection status according to sociodemographic information and water quality. A *P* value of 0.05 was considered significant.

Ethical clearance was obtained from the Institutional Ethical Committee of the Health Sciences Faculty of the University of Bangui. We respected the anonymity of the participants and the confidentiality of their information by using only the national identification code assigned to each PLHIV. Information on the purpose of the study was provided in both official languages ​​of the country (French and Sango), and informed consent was obtained before enrollment in the study. The results of the biological and molecular tests of each PLHIV participant were sent to their attending physician in a sealed envelope.

## RESULTS

The PLHIV comprised 156 women (78%) and 44 men (22%), for a sex ratio of 0.28 for men. The age ranged from 8 to 65 years (average, 38 years). Table 1 shows the seroprevalence by sex, age, and some risk factors for contamination with HEV. The overall seroprevalence of IgM anti-HEV in PLHIV in this study was 7.5% (8.9% females, 2.2% males), and the seroprevalence of IgG was 68% (48% females, 70.4% males). The IgM anti-HEV status of 5 people (2.5%; 2 men and 3 women) was indetermined. More men (70.4%) had IgG anti-HEV antibody than women (48%), and men had a higher risk of infection than women (OR, 2.4; 95% CI, 1.2–5.2; *P* = .016). PLHIV aged 30–36 years were more frequently positive than other age groups, but the difference was not statistically significant. No statistical difference was observed for studied risk factors. Table 2 shows the results of real-time RT-PCR of 50 samples of sera positive or indeterminate for HEV IgM and IgG antibodies. Amplification of 3 anti-HEV IgM and 3 anti-HEV IgG samples indicated that the viral genome was present. All samples that were serologically positive for anti-HEV IgM and >90% of the anti-HEV IgG-positive samples had normal alanine transaminase values. The presence of a recent HEV (positive IgM anti-HEV) infection seemed to have no influence on the TCD4+ lymphocyte count (Table 2). We also found no relation between hematological parameters and HEV infection (Table 3).

**Table 1.** Demographic Characteristics and Risk Factors for HEV Infection in 200 People Living With HIV

Characteristics	IgM Anti-HEV^a^				IgG Anti-HEV^b^			
	Positive, No. (%)	Negative, No. (%)	OR (95% CI)	*P*	Positive, No. (%)	Negative, No. (%)	OR (95% CI)	*P*
Prevalence	15 (7.5)	180 (90.0)			106 (53.0)	88 (44.0)		
Sex								
Female	14 (9.0)	139 (89.1)	-		75 (48.1)	76 (48.7)	-	
Male	1 (2.3)	41 (93.3)	4.1 (0.5–32.3)	.18	31 (70.5)	12 (27.3)	0.4 (0.2–0.8)	.01
Age, y								
≤29	4 (7.8)	44 (86.3)	-		23 (45.1)	24 (47.1)	-	
30–36	5 (9.3)	49 (90.7)	0.8 (0.01–50.0)	.9	28 (51.9)	25 (46.3)	0.8 (0.01–43.9)	.9
37–43	4 (8.7)	41 (89.1)	1 (0.01–0.62)	1.0	29 (63.0)	17 (37.0)	0.7 (0.01–41.7)	.9
≥44	2 (8.3)	21 (87.5)	1.8 (0.03–121.7)	.7	16 (66.7)	7 (29.2)	0.8 (0.02–46.4)	.9
Treated well water^c^								
No	1 (6.3)	15 (93.8)	-		10 (62.5)	6 (37.5)	-	
Yes	2 (12.5)	14 (87.5)	0.5 (0.04–5.73)	.5	9 (56.3)	6 (37.5)	1.1 (0.5–4.7)	.9
Distance between wells and latrine, m								
>30	9 (6.2)	133 (91.1)	-		76 (52.1)	65 (44.5)	-	
≤30	6 (11.1)	47 (87.0)	2.0 (0.2–24.1)	.6	30 (55.6)	23 (42.6)	0.4 (0.008–20.550)	.6

Abbreviations: CI, confidence interval; HEV, hepatitis E virus; OR, odds ratio.

^a^Five uncertain results for IgM anti-HEV.

^b^Six uncertain results for IgG anti-HEV.

^c^Only 32 patients have consumed water from wells.

**Table 2.** Serological Profile of HEV Infection in People Living With HIV

Infection Type	PCR^a^ and Serological Results			Nb	CD4, Mean	Alanine Transaminase, Mean
No contact	PCR-	IgM-	IgG-	0	-	-
Recent infection	PCR+	IgM-	IgG-	0	-	-
	PCR+	IgM+	IgG-	1	-	-
	PCR-	IgM+	IgG-	3	463	20
Recent infection	PCR+	IgM+	IgG+	3	573	13
	PCR-	IgM+	IgG+	6	439	24
Reinfection	PCR+	IgM-	IgG+	2	534	26
Past infection	PCR-	IgM-	IgG+	24	345	30

Abbreviations: HEV, hepatitis E virus; PCR, polymerase chain reaction; RT-PCR, reverse transcription polymerase chain reaction.

^a^Real-time RT-PCR.

**Table 3.** Hematological Parameters of People Living With HIV Infected by HEV

Blood Cells		IgM Anti-HEV		
		Positive, No. (%)	Indeterminate, No. (%)	Total
Red cells	<3.8 cells/mm^3^	5 (33.3)	1 (20)	60
	≥3.8 cells/mm^3^	10 (66.7)	4 (80)	140
Leukocytes	<5.5 × 10^3^ cells/mm^3^	6 (40)	4 (80)	111
	≥5.5 × 10^3^ cells/mm^3^	9 (60)	1 (20)	89
Platelets	<276 × 10^3^ cells/L	11 (73.3)	5 (100)	106
	≥276 × 10^3^ cells/L	4 (26.7)	0 (0)	94

Abbreviation: HEV, hepatitis E virus.

**Figure 1.**
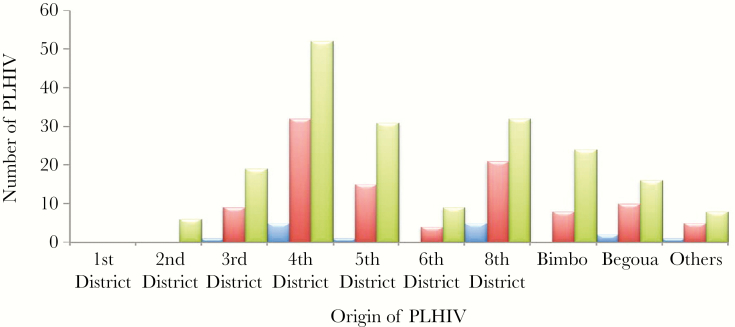
Residence of people living with HIV (PLHIV) patients with IgM–hepatitis E virus (HEV) antibodies (blue) and IgG-HEV antibodies (red) in Bangui and its suburbs. Green columns represent the total number of PLHIV in each district and suburbs. Others: districts or suburbs not determined.

Analysis of the samples by residence of PLHIV showed that IgM and IgG anti-HEV were detected predominantly in 5 districts of Bangui and in the city of Bégoua. Most patients who carried these 2 markers lived in the fourth, fifth, and eighth districts of Bangui (Figure 1).

## DISCUSSION

The overall seroprevalence of IgM anti-HEV in PLHIV in Bangui was 7.5% (8.9% females, 2.2% males), and the seroprevalence of IgG was 68% (48% females, 70.4% males). Although these values suggest that both sexes were exposed to HEV, our study confirms that males are more commonly exposed, as reported previously [16, 20, 21]. In addition, the seroprevalence rates obtained in the case of PLHIV are comparable to those obtained in the general population of Bangui after the 2004 outbreaks [17]. Although HEV infection has been reported to be more prevalent in young people [16], we found no significant difference in infection rates between young people and adults. The seroprevalence of both IgM and IgG HEV in PLHIV in our study is relatively comparable to that reported in Ghana [5] and Zambia [6]. However, it was relatively high compared to that reported in Cameroon [5], Gabon [22], and South Africa [23]. It should be noted that although HEV infection causes major hepatic injury and this virus circulates in sub-Saharan Africa, where HIV prevalence is high, coinfection between these 2 viruses has not been extensively studied. However, this variability between seroprevalence rates may be due to environmental conditions.

The high seroprevalence among the PLHIV in this study is probably due to the fact that HEV is endemic and epidemic in the country, and the prevalence of HIV is also high. Although the presence of IgG HEV antibodies in the population of Bangui has been reported since the mid-1990s, the first outbreaks of hepatitis E took place in Bangui in the early 2000s; since then, several outbreaks of hepatitis E have occurred in the Central African Republic [15–17]. Hygienic and sanitary conditions in the country are not optimal, and behavioral risk factors are of great importance [2, 4, 24]. Ingestion of viral particles by consumption of contaminated water, and more rarely by food soiled by human excreta, is probably the principal source of transmission.

We found no influence of HEV infection on transaminase levels, TCD4+ lymphocyte cells, or hematological parameters in PLHIV. Other authors have reported that HEV infection in PLHIV increases transaminase [25] and decreases TCD4+ cell levels [26–29]; however, our results are consistent with those of several other studies [5, 9, 14, 30, 31] that reported no elevation of transaminase levels or alteration of TCD4+ cell levels in PLHIV infected with HEV. The hematological parameters of the PLHIV included in our study did not appear to be altered by HEV coinfection, probably due to their therapeutic management, as other studies have shown that treatment with antiretrovirals can result in normalization of abnormally high levels of transaminases and stabilization of low levels of TCD4+ lymphocytes [30–32].

Using real-time RT-PCR, we demonstrated the presence of the virus in the population. It is often difficult to find HEV in blood samples because the viremia does not last long enough, although the virus can be excreted in the stools up to >6 months after infection [28]. Thus, the stools of all patients with HEV IgM should be analyzed for the virus to determine with certainty the presence of HEV. Unfortunately, we could not call back the patients to collect stool samples due to the armed conflict in the country.

We were successful in obtaining an acceptable number of people for the study from all 8 districts of Bangui and also from the suburbs of Bégoua and Bimbo. Sanitation service levels vary by district in Bangui. The first and second districts have operational sanitation services, whereas these services are deficient in the other 6 districts (third, fourth, fifth, sixth, seventh, and eighth), where sporadic epidemic cases have often occurred; they have also occurred in the suburb of Bégoua in the past [15–17]. Most of the PLHIV patients came from the fourth, fifth, and eighth districts of Bangui, and they presented more cases of HEV coinfection. These 3 districts often have overflowing drains and short-circuiting of networks of clean water and structures to purify wastewater. Latrines are often built close to wells, and the water is usually used without treatment. These insalubrious conditions might explain the presence of the virus and the high rate of infection by HEV of the PLHIV in these 3 districts. However, a larger study should be conducted to respond meaningfully to the many questions.

We were unable to identify the risk factors of PLHIV for infection with HEV. Although the statistical analysis suggests that the risk factors studied (consumption of untreated well water and distance from latrines to wells used by households) are not involved in the contamination of PLHIV, this does not preclude us from saying that the consumption of well water infected with HEV is the main source of contamination in the Central African Republic [4]. If consumption of nonpotable water is the main risk factor, we may have introduced bias by not testing pets and water from wells for the presence of HEV. It would also be useful to include more PLHIV to increase the statistical strength.

In conclusion, our results show that the seroprevalence of HEV is very high among PLHIV and in the general population of Bangui. It might be possible to monitor biochemical, biological, and hematological parameters in PLHIV by ensuring good therapeutic management of HEV and to extend the study of risk factors by isolating HEV from the stools of PLHIV and including samples from their environment. Our results should be taken into account when identifying the risk factors for HEV infection in PLHIV. The sample size may have been too small; larger studies are required to confirm these results.

## Supplementary Data

Supplementary materials are available at *Open Forum Infectious Diseases* online. Consisting of data provided by the authors to benefit the reader, the posted materials are not copyedited and are the sole responsibility of the authors, so questions or comments should be addressed to the corresponding author.
